# Relative risk of diabetes, dyslipidaemia, hypertension and the metabolic syndrome in people with severe mental illnesses: Systematic review and metaanalysis

**DOI:** 10.1186/1471-244X-8-84

**Published:** 2008-09-25

**Authors:** David PJ Osborn, Christine A Wright, Gus Levy, Michael B King, Raman Deo, Irwin Nazareth

**Affiliations:** 1 Department of Mental Health Sciences, (Royal Free Campus), University College London Medical School, Rowland Hill Street, London, NW3 2PF, UK; 2 Camden and Islington Mental Health and Social Care Trust, St Pancras Way, London, NW1 OPE, UK; 3 Department of Primary Care and Population Health, (Royal Free Campus) University College Medical School, Rowland Hill Street, London, NW3 2PF, UK; 4 MRC General Practice Research Framework, 158-160 North Gower Street, London NW1 2ND, UK

## Abstract

**Background:**

Severe mental illnesses (SMI) may be independently associated with cardiovascular risk factors and the metabolic syndrome. We aimed to systematically assess studies that compared diabetes, dyslipidaemia, hypertension and metabolic syndrome in people with and without SMI.

**Methods:**

We systematically searched MEDLINE, EMBASE, CINAHL & PsycINFO. We hand searched reference lists of key articles. We employed three search main themes: SMI, cardiovascular disease, and each cardiovascular risk factor. We selected cross-sectional, case control, cohort or intervention studies comparing one or more risk factor in both SMI and a reference group. We excluded studies without any reference group. We extracted data on: study design, cardiovascular risk factor(s) and their measurement, diagnosis of SMI, study setting, sampling method, nature of comparison group and data on key risk factors.

**Results:**

Of 14592 citations, 134 papers met criteria and 36 were finally included. 26 reported on diabetes, 12 hypertension, 11 dyslipidaemia, and 4 metabolic syndrome. Most studies were cross sectional, small and several lacked comparison data suitable for extraction. Meta-analysis was possible for diabetes, cholesterol and hypertension; revealing a pooled risk ratio of 1.70 (1.21 to 2.37) for diabetes and 1.11 (0.91 to 1.35) of hypertension. Restricting SMI to schizophreniform illnesses yielded a pooled risk ratio for diabetes of 1.87 (1.68 to 2.09). Total cholesterol was not higher in people with SMI (Standardized Mean Difference -0.10 (-0.55 to 0.36)) and there were inconsistent data on HDL, LDL and triglycerides with some, but not all, reporting lower levels of HDL cholesterol and raised triglyceride levels. Metabolic syndrome appeared more common in SMI.

**Conclusion:**

Diabetes (but not hypertension) is more common in SMI. Data on other risk factors were limited by poor quality or inconsistent research findings, but a small number of studies show greater prevalence of the metabolic syndrome in SMI.

## Background

People with severe mental illness (SMI) such as schizophrenia and bipolar affective disorder are at greater risk of coronary heart disease (CHD) than people without such diagnoses [1-3]. The mutable risk factors for CHD are smoking, hypertension, diabetes mellitus and high ratio of total cholesterol to High Density Lipoprotein (HDL) cholesterol. Although, many people with SMI are likely to be heavy smokers, and less likely to succeed in smoking cessation [4], the relationship between SMI and CHD mortality is not wholly explained by smoking[3] and there has been increasing interest in the prevalence of diabetes and dyslipidaemia in people with SMI. Second generation antipsychotics may exacerbate features of the metabolic syndrome including abnormal glucose and lipid profiles [2,5,6]. But recent reviews have suggested that people with SMI are at risk of the metabolic syndrome including diabetes irrespective of antipsychotic therapy [7,8]. People with SMI share other risk factors including unhealthy lifestyles [9] obesity and positive family histories [10].

We hypothesised that there were differences in the risk of abnormal glucose, blood pressure or lipid abnormalities between people with and without SMI. We searched for studies comparing the risk of diabetes or hyperglycaemia, hypertension, dyslipidaemia or a combination of these factors (as components of the metabolic syndrome or as an overall CHD risk score). We did not aim to assess smoking since a systematic review has recently been published [4] and the conclusions are uncontroversial.

## Methods

We searched for studies of diabetes or hyperglycaemia, hypertension, dyslipidaemia or combinations of these factors in people with and without SMI and systematically reviewed the literature to appraise the epidemiological evidence. We estimated the strength of any association between SMI and these CHD risk factors.

### Data sources and search strategy

We electronically searched MEDLINE, EMBASE, CINAHL, the Cochrane Library database & PsycINFO for articles in English, French, German, Italian or Spanish and sought papers published between 1897 and 2005 inclusively. We hand searched reference lists of review papers and made contact with authors and researchers to ensure comprehensive coverage. We piloted and modified our search strategy to retrieve all key papers in this field. The most sensitive search included three broad search themes namely 1) Terms related to SMI, 2) cardiovascular diseases and 3) the risk factors of diabetes, lipid disorders, hypertension, the metabolic syndrome and cardiovascular risk scores. Synonym lists were constructed for each theme and the databases were searched using these synonyms as both thesaurus and free-text terms (Additional file 1). For SMI, we included all terms relating to psychotic disorders, schizophreniform disorders, bipolar affective disorders and psychotic depression. Similarly all synonyms for search themes 2 and 3 were employed. We included an additional wider term for all mental disorders in a final search combined with both search themes 2 and 3. A combination of these two approaches provided the most reliable results.

### Study selection

We included cross sectional, case-control, cohort and intervention studies in which the risk factors of interest were available in a group with SMI and a reference group without SMI. We excluded pharmacological studies comparing CHD risk factors between different antipsychotics and without any comparison data from people not prescribed these drugs as these studies could not shed light on comparative risk between people with and without SMI. We included all studies involving representative groups with SMI and noted whether they were sampled from the community; outpatient settings, inpatients or from long stay psychiatric accommodation.

### Screening process

Two or more authors independently read all titles and available abstracts to identify potentially relevant articles. Decisions were compared and disagreements were discussed at steering group meetings involving all authors. We translated non-English articles to determine their relevance.

### Data extraction

We extracted data on the type of study design, the setting and the source of the groups with and without SMI. We recorded the type and method of SMI diagnosis and the reported response rate. We extracted which CHD risk factors (e.g. diabetes) were reported and how they were measured and defined. We noted whether all participants were screened for CHD risk or whether the outcome (e.g. diabetes) relied on screening and diagnosis being made during routine clinical care. Summary data (i.e. raw numbers and percentages) on the prevalence of risk factors were obtained for each group including raw numbers and percentages. Comparative statistics were noted including absolute differences in continuous outcomes or proportions and estimates of relative risk such as odds ratios. Adjustment of main results for confounders was also noted.

### Data synthesis

We defined three levels of evidence. The highest level were studies where a non-SMI comparison group was recruited. The next level were those that did not recruit a comparison group but used comparative risk factors data from general health population studies and the lowest level included studies where a selected group with other psychiatric diagnoses was used as a comparison. Within these levels we then grouped studies according to SMI diagnosis, and the sampling frame for the SMI group(e.g. community or from a specific secondary care setting such as an inpatient unit or clinic. Finally, where possible we calculated summary statistics such as risk ratios (RRs), confidence intervals and standardized mean differences (the mean difference in outcome/standard deviation for outcome; the effect size) for outcomes even when the papers had not presented such results. This was only possible when papers either reported raw numbers for dichotomous outcomes or means plus standard deviations for continuous outcomes.

### Meta-analysis

Data were entered into Stata version 9 [11] and standard meta-analytic techniques were employed if there were more than three studies for a given outcome. Meta-analyses could only conducted on studies that reported data from a comparison group. We calculated pooled estimates of effect sizes and risk ratios using a random effects model that uses inverse variance methods to apportion more weight to larger rather than smaller studies in the meta-analysis. We approached heterogeneity in results between studies in two ways. Firstly we assessed whether a significant level of difference existed using Mantel-Haenszel chi square tests. If the chi square test was significant below p = 0.05, we quantified the amount of heterogeneity using I^2 ^statistics. We considered I^2 ^above 50% as an indicative of substantial heterogeneity.

Where studies only reported percentages, we could only calculate risk ratios rather than odds ratios. For consistency we therefore present risk ratios rater than odds ratios in the meta-analyses.

## Results

**Figure 1:**
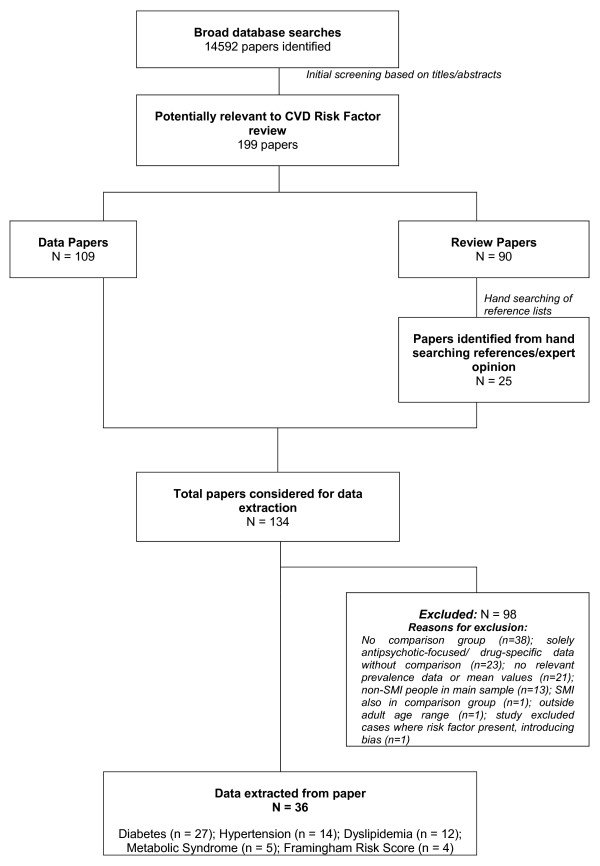
Flowchart of search results and studies included in final review.

The initial database search generated 14592 papers, 134 papers were identified for further scrutiny but more detailed assessment by up to four authors yielded 36 papers [12-47] that were eligible for inclusion in the final review (figure 1).

27 papers reported outcomes related to diabetes or hyperglycemia, 14 reported hypertension or blood pressure, 12 dyslipidaemia or lipid levels, 5 the metabolic syndrome and 4 papers included overall cardiovascular risk scores such as a ten year Framingham risk score [48]. Of the 98 excluded papers, the most common reason for ineligibility was that the paper only explored specific comparisons between different antipsychotic drugs, without comparison data on people not taking the drugs (n = 23). Many studies reported cardiovascular outcomes in samples of people with SMI without any reference data (n = 38). In several studies, samples included people with multiple diagnoses other than our definition of SMI (such as dementia), with no specific data for the subgroups with SMI as defined in this paper. Other reasons for ineligibility can be viewed in figure 1.

### Study characteristics

Of the 36 papers included, most (28/36) reported cross sectional data on one or more cardiovascular risk factors. There were 8 papers utilizing data from longitudinal studies [14-16,18,20,35,39,44], although strictly, none collected consecutive longitudinal information regarding cardiovascular risk factors at both baseline and follow-up. Four studies [12,13,33,45] recruited specifically from community settings, eight studies [14-16,24,25,37,46,47] from a mixture of outpatient and inpatient settings, and 5 studies [22,23,34,42,43] from outpatient clinics. The remaining 19 studies collected data from acute or long stay inpatient samples. These papers are summarized in additional file 2; tables 1–5, by outcome of diabetes/hyperglycaemia (additional file 2; table 1), hypertension/blood pressure (additional file 2; table 2), dyslipidaemia/lipid levels (additional file 2; table 3), metabolic syndrome (additional file 2; table 4) and 10 year CHD risk scores (additional file 2; table 5). Within these tables, the studies are grouped according to whether they recruited a comparison group or simply used general population data, and by source of the SMI sample (eg inpatients or community).

### Diabetes and hyperglycaemia

Twenty seven eligible papers [12-36,46,47] reported glucose related outcomes (additional file 2; table 1). The location of the studies, source, definition, SMI diagnosis and relevant outcomes for each study are summarized in additional file 2; table 1. Diabetes or hyperglycemia definitions included diagnosis or treatment for diabetes in the clinical records (n = 17) [12-18,22,24-27,30,34-36,46] self-reported diabetic diagnosis (n = 2) [23,25], screening results for random glucose (n = 2)[13,29] or fasting glucose(n = 3) [20,28,47] and impaired glucose tolerance (n = 3) [19,31,32]. Most studies (n = 23) included people with diagnoses of schizophrenia and/or schizoaffective disorder, two studies also included people with bipolar affective disorder [27,46]. Three studies only included people with a diagnosis of bipolar disorder [26,30,35].

**Figure 2:**
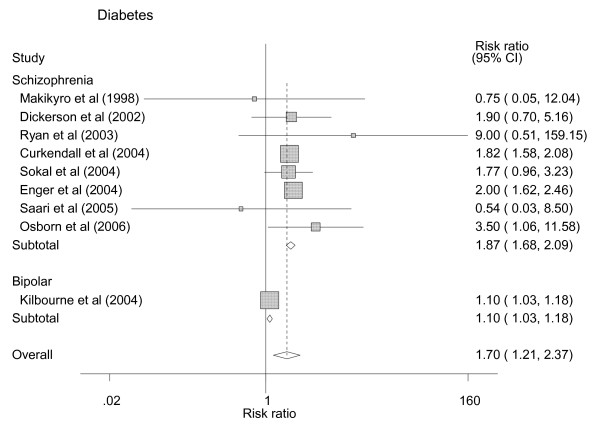
Random Effects Meta-Analysis on Risk Ratios of Diabetes Prevalence between those with schizophrenia or Bipolar Disorder and a control group.

Nine studies provided data that could be used in the meta-analysis for diabetes [12-16,18-20,23] (figure 2).

These studies involved 9612 people with SMI, 1166 of whom had a diagnosis of diabetes and a total of 3449677 people without SMI of whom 534248 had recognized diabetes. The pooled risk ratio for diabetes in SMI was 1.70 (1.21 to 2.37). There was considerable heterogeneity with a significant overall test for heterogeneity (chi square = 57.91 p < 0.001; I^2 ^= 91.2%). However, within the schizophrenia and/or schizoaffective disorder group there was no significant heterogeneity (p = 0.837, I^2 ^< 0.1%). In this group risk ratios for recorded diabetes ranged from 0.54 to 9.0 and the pooled risk ratio was 1.87 (1.68 to 2.09). The derived risk ratio from the one study including participants with bipolar affective disorder was 1.10 (1.03 to 1.18). There was no significant difference in results from studies of inpatient SMI samples compared to community samples (test for heterogeneity between subgroups p = 0.851).

**Figure 3:**
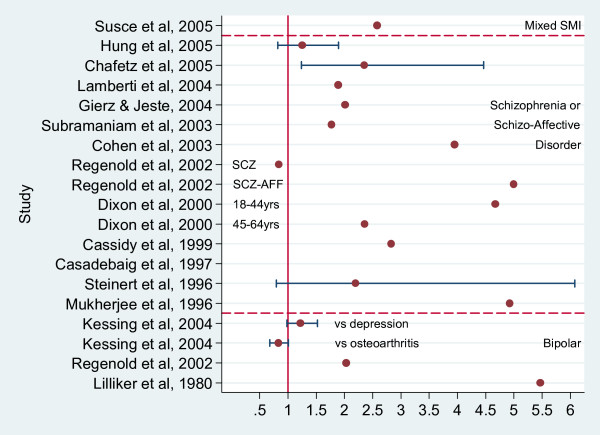
**Risk Ratios for Diabetes comparing those with Schizophrenia, Bipolar disorder or SMI with a comparison group.** Studies not suitable for inclusion in metaanalysis.

Figure 3 displays risk ratios for diabetes in SMI and, where possible, confidence intervals (exact figures in additional file 2; table 1). These are based on studies in which the data were unsuitable for inclusion in meta-analysis (e.g. comparison data only reported as percentages or using general population statistics without raw data).

Four studies compared random[13] or fasting[19,20,47] glucose levels, of which two [13,20] showed significantly increased standardized mean differences in the SMI group. The CATIE study [47] reported mixed results which differed by gender. Mean fasting glucose was significantly raised in SMI females but not males. However males with SMI were significantly more likely to reach criteria for raised fasting glucose than controls but this finding was not repeated in females (additional file 2; table 1)

### Hypertension

Fifteen papers reported data relating to hypertension (additional file 2; table 2). Most included people with schizophrenia or schizoaffective disorder but three papers included people with bipolar affective disorder P[16,38,46]. Hypertension was variously assessed by: self-report, existing use of anti-hypertensive medication, diagnosis of hypertension in clinical records, direct measurement of blood pressure and use of varying systolic and diastolic thresholds for hypertension (additional file 2; table 2).

**Figure 4:**
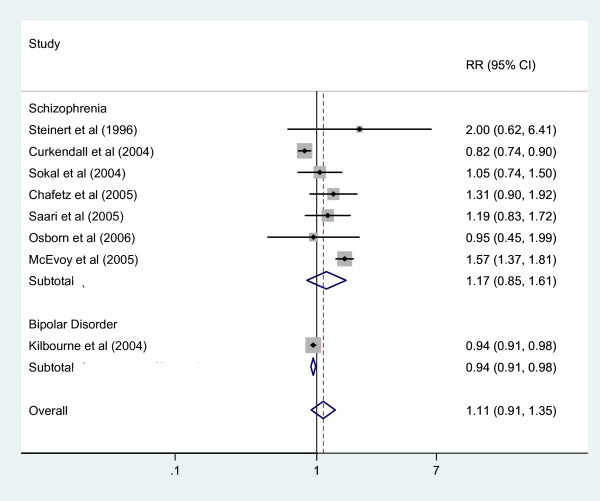
Random Effects Meta-analysis of Risk Ratio for prevalence of Hypertension between those with Schizophrenia, Bipolar or SMI and a Comparison Group.

Seven studies that included 2333/6249 people with SMI who were hypertensive and 1261228/2169371 hypertensive people without SMI were included in the meta-analysis and none showed significantly elevated risk for hypertension in the SMI group (figure 4). The pooled risk ratio for hypertension in SMI was 1.11 (0.91 to 1.35). Heterogeneity was significant (Chi square test p < 0.001 I^2 ^= 89.2%).

We were able to calculate risk ratios (but not confidence intervals) for hypertension from four further studies which also reported general population comparison data, resulting in two raised RRs [23,38] and two reduced RRs [34,46] for hypertension in SMI (additional file 2; table 2).

### Dyslipidaemia

**Figure 5:**
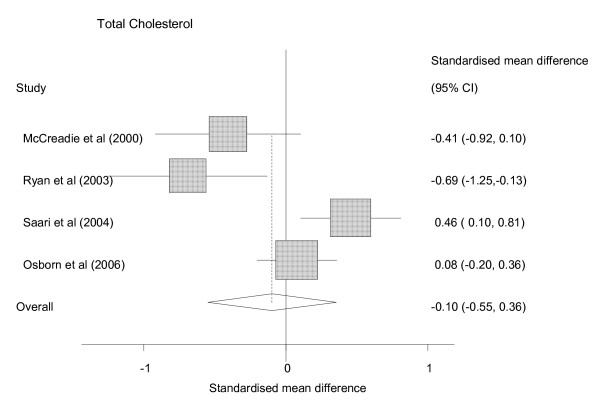
Random effects Meta Analysis of Studies reporting total cholesterol results for those with SMI and those without.

12 studies reported a variety of lipid outcomes including total cholesterol, High Density Lipoprotein (HDL) cholesterol and Low Density Lipoprotein (LDL) cholesterol and triglycerides (additional file 2; table 3), some using fasting samples and some not. Seven included raw data from a comparison group, and only three studies included people with bipolar affective disorder. The only lipid outcome reported in sufficient studies for meta-analysis was mean total cholesterol (figure 5). Total cholesterol values were available for 160 people with SMI and 5702 people without SMI in 4 different studies [13,19,39,40] The pooled SMD was -0.10 (0.55 to 0.36) (figure 5). There was significant heterogeneity; (chi square = 14.91, p = 0.002; I^2 ^= 79.9%) with one study showing an SMI group with lower total mean cholesterol and another showing SMI the opposite result.

**Figure 6:**
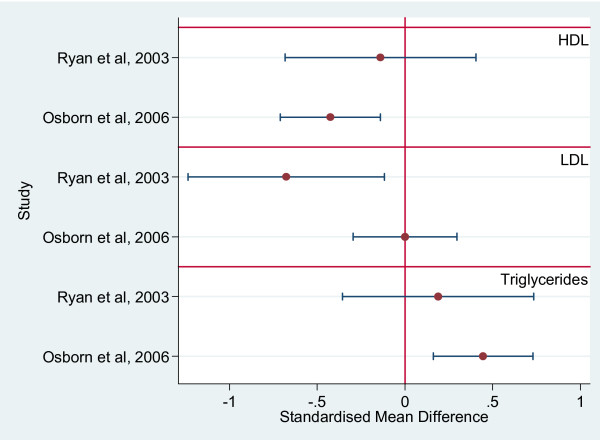
Plot of Standardised Mean Difference between those with Schizophrenia and a control group for HDL, LDL and Triglycerides.

Standardized mean differences between SMI and non-SMI samples could be calculated for HDL cholesterol, LDL cholesterol and triglycerides in two studies [13,19] (figure 6).

One of these studies found significantly lower HDL levels and higher triglyceride levels [13]. The other found significantly lower LDL levels [19] (figure 6).

Some studies also reported lipid results based on: a diagnosis of hyperlipidaemia or lipid disorders (by ICD criteria) [14,16] receipt of antilipemic medication[14], or proportions of people with lipid levels exceeding a defined threshold [13,20,47]. These results were inconsistent. In some studies people with SMI were significantly more likely to have low HDLP[13,47] high triglycerides[20,47] or a high HDL/total cholesterol ratio but in others these did not reach significance for either HDL[20] and LDL levels[13] or total cholesterol [13]. A calculated risk ratio for prevalence of ICD 9 dyslipidaemic disorders was not significant (0.86: 0.73 to 1.01) [14].

Studies with insufficient data for calculating SMDs, or where other patient groups were used for comparison, also reported conflicting results. SMI was associated with significantly lower total cholesterol in one study[36] with significantly lower HDL cholesterol in others [37,47] but this was not confirmed elsewhere[39]. Triglyceride results were also inconsistent [39,41,47].

### Metabolic syndrome

**Figure 7:**
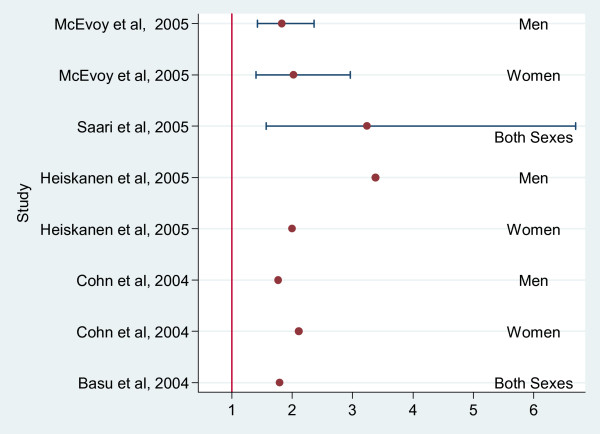
Risk Ratios for Metabolic Syndrome comparing those with Schizophrenia, SMI or Bipolar disorder to a control group or general population.

Five papers involving 1026 people with schizophrenia or schizoaffective disorder reported prevalence of the metabolic syndrome according to international criteria, but only two studies used raw data from a comparison group in addition to 718 people with schizophrenia [20,47] (additional file 2; table 4). The other three studies used general population comparison data that were not suitable for meta-analysis (due to a lack of raw numbers) and not clearly age matched to the people with SMI. Figure 7 displays risk ratios (and where possible calculated confidence intervals) from all five studies. All point estimates for risk of the metabolic syndrome were raised.

### Ten year cardiovascular risk scores

Four studies report ten year cardiovascular risk scores for people with SMI, two included comparison groups [13,44], and two used general population comparison data [37,45] (additional file 2; table 5). In two studies, involving 352 people with SMI, the 10 year cardiovascular risk scores was significantly increased in men but not women with SMI [37,45]. One study of 21 first-onset cases of schizophreniform illnesses showed significantly raised 10 year risk scores compared to general population, but no differences compared to matched controls [44]. Finally, one controlled community study found that excess cardiovascular risk scores were only detectable when different effects were considered at different age groups [13].

## Discussion

We found that diabetes mellitus is the cardiovascular risk factor most convincingly associated with SMI. Meta-analysis of the highest quality studies revealed almost a two-fold risk of diabetes in schizophrenia-like illnesses but not bipolar affective disorder. Conversely, meta-analysis revealed no association between SMI and hypertension. Similar findings were observed for total cholesterol levels, but these studies were limited by their design and so conclusions must be guarded. There were inadequate numbers of comparative studies of other lipids, such as HDL cholesterol, or of the metabolic syndrome to conduct a meta-analyses. Lower HDL cholesterol levels in people with SMI found in two studies [13,47] were not confirmed by another [20]. Five studies on the metabolic syndrome revealed an excess risk in people with SMI, and two, involving 718 people with SMI, confirmed such an excess of metabolic syndrome statistically. Raised "Framingham" or ten year cardiovascular risk scores may only be demonstrable in SMI when differences in effects are examined separately in different age groups and sexes. For instance excess risk scores may only be detectable in those over 40.

### Quality and variability of published studies

There were very few high quality comparative studies on cardiovascular risk factors and the metabolic syndrome in people with and without SMI. Many studies in this review were limited by small, convenience samples of people with SMI such as those in specific clinics or inpatient units, compromising the generalisability of their findings. Furthermore, several studies reporting higher levels of cardiovascular risk in SMI did not obtain raw comparison data within their study to allow statistical assessment of the importance of their findings. Cardiovascular risk factors are increasing rapidly in the general population, hence the need for relevant contemporary comparison figures. There were no studies designed to compare the longitudinal development of cardiovascular risk factors between people with and without SMI.

The meta-analysis for diabetes did not detect any heterogeneity between inpatient and community samples with schizophreniform illnesses, suggesting consistency between settings despite the sampling bias inherent to inpatient samples. However the result for people with bipolar disorder was significantly different from that for schizophrenia (figure 2). The marked heterogeneity score from the hypertension meta-analysis suggested that there was considerable variation between studies which may have arisen from the differing sampling methods and/or different definitions of hypertension employed in different papers.

Based on our second level of evidence, (namely studies utilizing general population figures for comparison data) the estimated excess risk of diabetes varied between a zero and a fivefold risk (figure 3). Only three of these studies permitted calculation of confidence intervals for diabetes risk ratios and two of these were not significant at the 5% level (figure 3). This wide variation in the magnitude of diabetes risk between studies may reflect differences in 1) the sampling and definition of SMI, 2) the source of the comparison group (and their inherent risk for diabetes), and 3) the definition of diabetes or hyperglycemic outcomes. Furthermore, studies that rely on identification of cardiovascular risk factors in routine clinical practice may be flawed due to differential screening rates in people with and without SMI. In the past, people with SMI may have been less likely to be screened for diabetes. More recently there is evidence that people prescribed certain second generation antipsychotics are more likely to receive screening for diabetes. The direction of bias due to differential screening rates may therefore extend in either direction, leading to underestimation or overestimation of diabetes prevalence in SMI, compared to people without.

This review included several small studies that may have lacked statistical power to detect real differences in risk factors or the metabolic syndrome. Few studies have investigated effect modification by age, but there is some support of this phenomenon when comparing people with and without SMI [3,13].

### Strengths and limitations

This is the first review to systematically appraise quality and synthesise data from comparative studies of diabetes, hypertension and lipid levels in people with and without SMI.

We paid critical attention to the quality of studies, in terms of the representativeness of samples, the outcomes measured and we present a large volume of comparative results regarding the prevalence of cardiovascular risk in SMI.

We have grouped these studies according to levels of quality within additional file 2; tables 1–5, especially regarding their selection of comparison data and where possible explored the role of different diagnoses and sampling methods in the meta-analysis. There were insufficient papers to allow us to further subdivide the results.

The review was labour intensive, and like other systematic reviews there was inevitably a delay between the search and publication of the review. The search strategy retrieved over 14,000 papers. However narrowing the search terms was not acceptable because the restricted search missed several important papers of which we were aware.

Therefore, during the production of this review further evidence may have emerged subsequent to our original search. From our knowledge of the field we are aware of one quality paper meeting our criteria involving both SMI and controls which was published in 2007. Mackin et al [49] reported data consistent with the main findings of this review. They compared metabolic parameters in 90 people with severe mental illnesses and 92 without. They report increased rates of cardiovascular risk factors in SMI including impaired glucose metabolism, lower HDL cholesterol and raised LDL cholesterol, raised triglycerides and increased metabolic syndrome. In common with our findings, blood pressure was not raised in this study [49].

These recent results have not been included in our analysis because this would bias the systematic nature of our review. Legitimate inclusion of this paper would require re-running of the search for other papers from 2007 and reviewing potentially thousands of new titles. This would be beyond the scope of our current funding.

We acknowledge the difficulty of synthesizing data from multiple studies. In this field many existing studies of cardiovascular risk factors have been opportunistic and have not ensured their SMI samples are representative nor that comparison data are comparable in terms of ethnicity and socio-economic deprivation. This may further explain the observed variation in results and heterogeneity. Furthermore several papers which are commonly cited as evidence for increased cardiovascular risk in SMI could not be included as they contained insufficient data or no comparison data.

In particular, studies that rely on clinical diagnoses for outcome definition are problematic, since many people may have cardiovascular risk which is undetected (such as abnormal lipids). Screening for lipids, blood pressure and glucose probably occurs in less than a third of people with SMI during routine practice [50]. These points may explain why other narrative reviews conclude the risk of diabetes may be even higher in people with SMI [7,8].

A further challenge is the employment of different definitions of outcomes such as diabetes, hypertension and metabolic syndrome in different studies. We minimised this problem by only including papers which compare risks in people with and without SMI using the same definition, thus focusing on the *relative *rather than *absolute *risk. The relative risk is less sensitive to the employment of differing definitions.

The poor epidemiological quality of studies in this field has been highlighted by a complementary systematic review [51] examining the relative diabetogenic risk of first and second generation antipsychotics. The authors found methodological weaknesses in most studies and were only able to make tentative conclusions about the possible role of second generation antipsychotics in the aetiology of diabetes.

### Explanation of excess risk

No studies longitudinally assessed predictors of diabetes (or other cardiovascular risk factors) in SMI. The relative contribution of second generation antipsychotics [2,4,6], lifestyle[10], family history [10], social deprivation and SMI itself (perhaps through chronic stress models) are still debated [7]. This possible role of SMI itself is supported by two small studies suggesting metabolic disturbances may be observable in newly diagnosed or drug naïve people with SMI [52,53]. However our findings reveal that we do not have an accurate estimate of the contribution of either second generation antipsychotics or SMI itself, to support or refute these theories. We know that the relative risk of metabolic harm differs between the second generation antipsychotics [2,4,6,10] but the absolute attributable risks are unclear.

To understand excess cardiovascular disease in SMI we must improve our knowledge of cardiovascular risk factors in two areas. First, we must accurately determine the extent of the excess of diabetes, dyslipidaemia, hypertension and the metabolic syndrome in representative samples of people with and without SMI, taking into account the effects of age, gender and socio-economic status. This can be achieved in studies with adequate comparison data that explore the level of risk apportionable to SMI and to relevant confounders, especially socio-economic status [13]. Secondly, to determine the risk attributable to antipsychotic medication, lifestyle, stress and addictions to cardiovascular risk we require data from carefully designed prospective studies. Retrospective estimates of such exposures in SMI are limited by inaccurate reporting and recall bias. Finally, these studies should be adequately powered and should test specific mechanistic hypotheses, rather than measuring multiple risk factors.

## Conclusion

Our findings emphasise the importance of poor physical health outcomes in people with SMI, including adverse cardiovascular outcomes. However, we have highlighted gaps in our current knowledge base. This review suggests that diabetes is indeed more common in SMI. Metabolic syndrome may also be more common, while there is far weaker evidence regarding dyslipidaemia and hypertension. We require high quality studies in representative samples of people with SMI in which all participants have been screened for cardiovascular risk. These should be cross-referenced to contemporary comparison data regarding the incidence of risk factors in the general population. Furthermore studies should explore differences in risk factors at different ages and require the statistical power to do so.

The mechanism underlying adverse cardiovascular outcomes remains poorly understood and it is premature to quantify the roles of antipsychotic medication, social adversity, psychiatric symptoms, physiological stress, smoking and diet on the causal pathway of cardiovascular diseases.

## Abbreviations

CHD: Coronary Heart Disease; HDL: High density lipoprotein (cholesterol); LDL: Low density lipoprotein (cholesterol); RR: Relative Risk; SMD: Standardised mean difference; SMI: Severe mental illness.

## Competing interests

The authors declare that they have no competing interests.

## Authors' contributions

DO, IN and MK had the original idea for the study. The protocol was designed and refined by them along with CW and GL. The papers and data for the review were retrieved, reviewed and analysed by all authors including RD. GL led the analysis DO and CW wrote the initial draft and tables. All authors commented substantially on subsequent versions of the manuscript and all authors have approved the final version.

## Funding

The study was funded by a Trial Platform grant from the UK Medical Research Council. Reference: G0301032

## Pre-publication history

The pre-publication history for this paper can be accessed here:



## Supplementary Material

Additional file 1 Search terms. More detail regarding search strategy and terms employed. Click here for file

Additional file 2 Results Tables 1–5. All papers included in final review are detailed with authorship, date, description of samples, outcomes measured and main results including summary statistics. These table are included as an additional file in accordance with journal style allowing us to maximise readability by presenting the tables in landscape. Click here for file
