# Supplementary material for: Relative risk of diabetes, dyslipidaemia, hypertension and the metabolic syndrome in people with severe mental illnesses: Systematic review and metaanalysis
Source: BMC Psychiatry. 2008 Sep 25;8:84. doi: 10.1186/1471-244X-8-84 (PMC2570660; doi:10.1186/1471-244X-8-84)
Supplement: Additional file 1 — Search terms. More detail regarding search strategy and terms employed. [file 1471-244X-8-84-S1.pdf]

Search terms for Osborn et al: Relative risk of diabetes, dyslipidaemia, hypertension and the metabolic syndrome in people with severe mental illnesses.
Systematic review and metaanalysis

Appendices

Three groups/concepts were developed to execute the search to maximum effectiveness in terms of sensitivity and specificity and these are detailed below

SMI terms

((BIPOLAR DISORDER* in TI,AB,MA) or (psychotic symptom* in TI,AB,MA) or (PSYCHOTIC ILLNESS* in TI,AB,MA) or (explode "Schizophrenia-" / all SUBHEADINGS in MIME,MJME) or ("Schizophrenia-Paranoid" / all SUBHEADINGS in MIME,MJME) or (SCHIZOPHRENI* in TI,AB,MA) or ("Psychotic-Disorders" / all SUBHEADINGS in MIME,MJME) or (SEVERE ENDURING MENTAL* in TI,AB,MA) or (severe mental illness* in TI,AB,MA) or (PSYCHOTIC DISORDER* in TI,AB,MA) or (SCHIZO AFFECTIVE* in TI,AB,MA) or (SCHIZOAFFECTIVE) or (SCHIZOAFFECTIV* DISORDER* in TI,AB,MA) or (MANIC* DEPRESS* in TI,AB,MA) or ("Bipolar-Disorder" / all SUBHEADINGS in MIME,MJME))

(mental disorder* in TI,AB,MA)* or ("Mental-Disorders" / all SUBHEADINGS in MIME,MJME)*

Cardiovascular terms

(myocardial ischaemi* in TI,AB,MA) or ((CORONARY HEART DISEASE) or (TACHYCARDI* in TI,AB,MA) or (explode "Tachycardia-" / all SUBHEADINGS in MIME,MJME) or (LONG QT SYNDROME in TI,AB,MA) or ("Long-QT-Syndrome" / all SUBHEADINGS in MIME,MJME) or (ARRHYTHMI* in TI,AB,MA) or (explode "Arrhythmia-" / all SUBHEADINGS in MIME,MJME) or (HEART DISEASE* in TI,AB,MA) or ("Heart-Diseases" / all SUBHEADINGS in MIME,MJME) or (cardiovascular disease* in TI,AB,MA) or ("Cardiovascular-Diseases" / all SUBHEADINGS in MIME,MJME) or (HEART ATTACK* in TI,AB,MA) or ("Heart-Arrest" / all SUBHEADINGS in MIME,MJME) or (HEART ARREST* in TI,AB,MA) or ("Myocardial-Infarction" / all SUBHEADINGS in MIME,MJME) or (MYOCARDIAL INFARCTION* in TI,AB,MA) or ("Coronary-Disease" / all SUBHEADINGS in MIME,MJME) or (coronary disease* in TI,AB,MA) or (myocardial ischemia in TI,AB,MA) or (MYOCARDIAL DISEASE* in TI,AB,MA) or ("Myocardial-Diseases" / all SUBHEADINGS in MIME,MJME))

Terms related to cardiovascular risk factors, and physical comorbidity

Hypertension
(high blood pressure in TI,AB,MA) or (hypertensi* in TI,AB,MA) or (explode "Hypertension-" / all SUBHEADINGS in MIME,MJME)
Hyperlipidemia
(hyperlipid* in TI,AB,MA) or ((explode "Hyperlipoproteinemia-+" / all SUBHEADINGS in MIME,MJME) or (explode "Hypercholesterolemia-+" / all SUBHEADINGS in MIME,MJME) or (explode "Hypertriglyceridemia-" / all SUBHEADINGS in MIME,MJME) or (explode "Hyperlipidemia-" / all SUBHEADINGS in MIME,MJME)) or (lipid* in AB,TI,MA) or (cholesterol in TI,MA,AB) or (explode "Cholesterol-" / all SUBHEADINGS in MIME,MJME)
Diabetes

(treatment emergent diabet* in TI,AB,MA) or (impaired glucose metabolism in TI,AB,MA) or (impaired glucose tolerance in TI,AB,MA) or (diabet* in TI,AB,MA) or (("Diabetes-Mellitus-Type-II" / all SUBHEADINGS in MIME,MJME) or ("Diabetes-Mellitus" / all SUBHEADINGS in MIME,MJME))

Left Ventricular Hypertrophy

(left ventricular hypertrophy in TI,AB,MA) or (explode "Hypertrophy-Left-Ventricular" / all SUBHEADINGS in MIME,MJME)

Smoking

("Smoking-Cessation" / all SUBHEADINGS in MIME,MJME) or (smoking in TI,AB,MA) or (explode "Smoking-" / all SUBHEADINGS in MIME,MJME) or (tobacco smoking in TI,AB,MA) or ("Tobacco-Use-Disorder" / all SUBHEADINGS in MIME,MJME) or (cigarette* in TI,AB,MA) or (explode "Tobacco-Use-Cessation" / all SUBHEADINGS in MIME,MJME)

Diet

(nutrition in TI,AB,MA) or (explode "Nutrition-" / all SUBHEADINGS in MIME,MJME) or (explode "Food-Habits" / all SUBHEADINGS in MIME,MJME) or (explode "Obesity-" / all SUBHEADINGS in MIME,MJME) or (diet in TI,AB,MA) or ((explode "Diet-Atherogenic" / WITHOUT SUBHEADINGS in MIME,MJME) or (explode "Diet-" / all SUBHEADINGS in MIME,MJME))

Exercise

(explode "Exercise-" / all SUBHEADINGS in MIME,MJME) or (physical activity in TI,AB,MA) or (exercise in TI,AB,MA)

Self-Care

(explode "Self-Care" / all SUBHEADINGS in MIME,MJME) or (self care in TI,AB,MA)

Terms excluded due to excessive yield of irrelevant papers.

“Risk”

(risk factor* in TI,AB,MA) or (explode "Risk-" / all SUBHEADINGS in MIME,MJME)

Antipsychotics

(neuroleptic* in TI,AB,MA) or (psychotropic* in TI,AB,MA) or (explode "Psychotropic-Drugs" / all SUBHEADINGS in MIME,MJME) or (atypical antipsychotic* in TI,AB,MA) or (explode "Antipsychotic-Agents" / all SUBHEADINGS in MIME,MJME)

Alcohol

(alcohol* in TI,AB,MA) or ((explode "Drinking-" / all SUBHEADINGS in MIME,MJME) or (explode "Alcoholism-" / all SUBHEADINGS in MIME,MJME) or (explode "Alcohol-Drinking" / all SUBHEADINGS in MIME,MJME) or (explode "Drinking-Behavior" / all SUBHEADINGS in MIME,MJME))

Attitude to health/health promotion

((explode "Health-Promotion" / all SUBHEADINGS in MIME,MJME) or (explode "Life-Style" / all SUBHEADINGS in MIME,MJME) or (explode "Health-Behavior" / all SUBHEADINGS in MIME,MJME)) or (health behavio* in TI,AB,MA) or (attitude to health in TI,AB,MA) or (attitude to health) or (("Health-Knowledge-Attitudes-Practice" / WITHOUT SUBHEADINGS in MIME,MJME) or ("Attitude-to-Health" / all SUBHEADINGS in MIME,MJME) or ("Patient-Acceptance-of-Health-Care-+" / all SUBHEADINGS in MIME,MJME))
