# Supplementary material for: Relative risk of diabetes, dyslipidaemia, hypertension and the metabolic syndrome in people with severe mental illnesses: Systematic review and metaanalysis
Source: BMC Psychiatry. 2008 Sep 25;8:84. doi: 10.1186/1471-244X-8-84 (PMC2570660; doi:10.1186/1471-244X-8-84)
Supplement: Additional file 2 — Results Tables 1–5. All papers included in final review are detailed with authorship, date, description of samples, outcomes measured and main results including summary statistics. These table are included as an additional file in accordance with journal style allowing us to maximise readability by presenting the tables in landscape. [file 1471-244X-8-84-S2.pdf]

TABLE 1: DIABETES AND HYPERGLYCAEMIA PAPERS

Source	Patient sample description	Comparison group description	Outcome measure	Prevalence estimates or Mean/SD	Main results
(A) STUDIES WITH A CONTROL GROUP					
Community Samples					
Sokal et al, 2004<br>(USA) [12]	N = 100. Men and women with SCZ or S-AFF	N = 3052. Matched for age/race/gender (15:1 ratio). From NHIS national survey dataset	Diabetes (ascertained from patient interview) – lifetime prevalence	*SMI:* Obs = 10/97 (10.3%)<br>*Control:* Obs = 167/2861 (5.8%)	Unadjusted OR = 1.98 (0.99 to 3.96);<br>Adjusted OR[c] = 1.98 (0.95 to 4.10)<br>*Calculated RR = 1.77 (0.96 to 3.23)
Osborn et al, 2006<br>(UK: England) [8]	N = 74. Men and women with SCZ, S-AFF or N-ACP	N = 148. Men and women	Diabetes (recorded in GP records)<br><br><br><br>Hyperglycaemia (random blood glucose > 11.0 mmol/L)<br><br><br>Mean random glucose levels (mmol/L)	*SMI:* Obs = 7/74 (9.6%)<br>*Control:* Obs = 4/148 (2.7%)<br><br><br>*SMI:* Obs = 5/74 (6.9%)<br>*Control:* Obs = 6/148 (4.1%)<br><br>*SMI:* Mean = 6.1 mmol/L (SD 3.5)<br>*Control:* Mean = 5.3 mmol/L (SD 2.1)	Unadjusted OR = 3.8 (1.1 to 13.3);<br>Adjusted OR[a] = 3.7 (0.9 to 15.4);<br>Adjusted OR[b] = 6.0 (1.2 to 3.10)<br>*Calculated RR = 3.50 (1.06 to 11.58)<br>Unadjusted OR = 1.7 (0.5 to 5.9);<br>Adjusted OR[a] = 1.2 (0.3 to 4.9);<br>Adjusted OR[b] = 1.1 (0.2 to 5.4)<br>SMI > Controls: t-test:: F = -2.1, p = .003<br>*SMD= 0.302 (0.02-0.583)
McEvoy et al, 2005<br>(USA) [47]	N=689. Men and women with chronic SCZ	Compared to randomly selected population from the NHANES III study matched for age/race/gender (N=689)	1). Mean fasting glucose (mg/dl) (no SD)<br>2). Met the glucose criterion for metabolic syndrome (>110mg/dl)	*Males:SMI mean =97.7, Control mean=102.4*<br>*Females: SMI mean=100.9 Control mean= 99.9*<br>*Males: SMI 14.1%, Controls 14.2%*<br>*Females SMI 21.7%, Controls 11.2%*	SMI<controls: t test p=0.034<br>SMI=controls: t test p=0.793<br>SMI=controls: chi sq p=0.964<br>SMI>controls: chi sq p=0.008
Outpatient and Inpatient Samples					
Curkendall et al, 2004<br>(USA) [14]	N = 3022. Men and women with SCZ. Members of US health plans	N = 12088. Matched for age/gender (4:1 ratio). Randomly selected from same health databases	Diabetes diagnosis (ICD-9 Code 250) *or* prescriptions for oral anti-diabetic medications or insulin	*SMI*: Prevalence = 91.7 per 1000<br>Incidence = 7.0 per 1000 person-years<br>*Control*: Prevalence = 50.5 per 1000<br>Incidence = 4.3 per 1000 person-years	*Prevalence*: Unadjusted OR = 1.9 (1.6 to 2.2);<br>Adjusted OR[d] = 2.1 (1.8 to 2.4)<br>*Incidence:* Unadjusted RR = 1.6 (1.2 to 2.2);<br>Adjusted RR[d] = 1.8 (1.2 to 2.6)<br>*Calculated risk ratio = 1.82 (1.58 to 2.08)
Enger et al, 2004<br>(USA) [8]	N = 1920. Men and women with SCZ. Members of US health plan	N = 9600. Matched for age/gender (5:1 ratio). Identified from same health plan database	Diabetes diagnosis (ICD-9 Code 250) *or* at least one dispensing of anti-diabetic drug	*SMI*: Obs = 105 events<br>Rate = 3464 per 100,000 person-years<br>*Control:* Obs = 201 events<br>Rate = 1380 per 100,000 person-years	Adjusted rate ratio[e] = 1.75 (1.38 to 2.21)<br>*Calculated risk ratio = 2.00 (1.62 to 2.46)
Kilbourne et al, 2004<br>(USA) [16]	N = 4310. Men and women with BPAD from National US Veterans cohort	N = 3408760. Men and women. From same Veterans cohort	Diabetes diagnosis (ICD-9)	*SMI:* Obs = 743 /4310 (17.2%)<br>*Control:* Obs = 532926/3408760 (15.6%)	None reported<br>*Calculated risk ratio = 1.10 (1.03 to 1.18)
Inpatient Samples					
Finney, 1989<br>(Sweden) [17]	Search of inpatient register (1969-1983) to identify conscripts (N = 621074) with diabetes diagnosis *and* SCZ. All males aged under 27 years	Members of same conscript cohort (National Enrolment Register 1969-1979) with diabetes diagnosis but *no* SCZ. All males aged under 27 years	*Juvenile* onset diabetes (ICD Code 250)	*Diabetics with schizophrenia: Obs* = 0; *Exp* = 4<br>*Diabetics without schizophrenia:*<br>*Obs* = 1154 cases (Rate = 1.9 per 1000)<br>*General population rates (1969-1983):*<br>For age 0-19 = 0.2 per 1000	None reported
Makikyro et al, 1998<br>(Finland) [18]	N = 89. Members of 1966 Birth Cohort with SCZ. Followed up 1982-1994. No gender information	N = 10630. Members of same Birth Cohort with no history of psychiatric disorder. Men and women	Diabetes mellitus (hospital-treated cases): ICD-8 and ICD-9	*SMI:* Obs = 0/89 (0%)<br>*Control:* Obs = 78/10630 (0.7%).	None reported<br>*Calculated risk ratio = 0.75 (0.05 to 12.04)
Ryan et al, 2003<br>(Finland) [19]	N = 26. Men and women with SCZ. First-episode and drug-naïve	N = 26. Men and women	Impaired glucose tolerance (>110 mg/dl and <125 mg/dl)<br>Mean fasting blood glucose (mg/dL)	*SMI:* 4/26 (15.4%)<br>*Control:* 0/26 (0%)<br>*SMI:* Mean = 95.8 (SD 16.9)<br>*Control:* Mean = 88.2 (SD 5.4)	None reported<br>*Calculated risk ratio = 9.00 (0.51 to 159.15)<br>SMI > Controls (t-test, p <.03)<br>*SMD = 0.606 (0.049 to 1.162)
Saari et al, 2005<br>(Finland) [20]	N = 31. Men and women with SCZ. Members of 1966 Birth Cohort followed up in 1997/1998	N = 5455. Men and women with no history of psychiatric treatment. From same 1966 Birth Cohort	Fasting blood glucose $\geq$ 110 mg/dL	*SMI:* Obs = 0/31 (0%)<br>*Control:* Obs = 157/5455 (3%)	None reported<br>*Calculated risk ratio = 0.54 (0.03 to 8.50)
Arranz et al, 2004<br>(Spain) [21]	Men and women with SCZ.<br>*Antipsychotic-free group:* N=50<br>*Antipsychotic-naive group:* N = 50 (first psychotic episode)	N = 50. Men and women. Hospital staff with no history of SCZ or medication affecting glucose homeostasis	Mean fasting blood glucose (mmol/L)	*Antipsychotic-free:* Mean = 4.47 (SD 0.08)<br>*Antipsychotic-naïve:* Mean = 4.33 (SD 0.05)<br>*Control group:* Mean = 4.22 (SD 0.08)	No significant differences– ANCOVA[f], p = .21<br>*SMDs<br>*Antipsychotic-free* = 3.125 (2.538 to 3.712)<br>*Antipsychotic-naïve* = 1.649 (1.194 to 2.104)

TABLE 1: DIABETES AND HYPERGLYCAEMIA PAPERS (continued)

(B) STUDIES COMPARING PATIENTS WITH SMI TO NORMATIVE DATA					
Source	Patient sample description	Comparison group description	Outcome measure	Prevalence estimates or Mean/SD	Main results
Outpatient Samples					
Lamberti et al, 2004 (USA) [22]	N = 196. Patients with SCZ. No gender information	Compared to general population rate for comparable age groups (data from Mokdad et al, 2001)	Diagnosis of diabetes (medical chart review)	*SMI:* Obs = 27/196 (13.8%)<br>*General population rate:* 7.3%	None reported.<br>*Calculated risk ratio = 1.89
Dickerson et al, 2002 (USA) [23]	N = 43. All *females*, with SCZ or S-AFF	N = 101. Age-range matched females from Maryland Behavioural Risk Factor Survey (BFRS) 1999 dataset	Diabetes (ever) – self-reported in interview using BRFS tool	*SMI:* Obs = 6/43 (14%) (CI: 5 to 28%)<br>*Controls:* Obs = 8/101 (8%) (CI: 6 to10%)	None reported<br>*Calculated risk ratio = 1.90 (0.70 to 5.16)
Outpatient and Inpatient Samples					
Casadebaig et al, 1997 (France) [24]	N = 3470. Men and women with SCZ	Compared to INSEE-CREDES 1991 general population survey data.	Treatment for diabetes (ICD-9 Code 250)	*SMI:* Males Obs = 24; Females*:* Obs = 30<br>*Controls:* No prevalence estimates reported	*Standardised Morbidity Ratio*<br>Male SMR = 4.0 (Chi square p<.05)<br>Female SMR = 2.0 (Chi square p<.05).
Dixon et al, 2000 (USA) [25]	(i) *Field Study* (1994-96): N = 719. Men and women with SCZ. (ii) *Medicaid Data* (1991): N = 6066. Men and women with SCZ, S-AFF or SFD. (iii) *Medicare Data* (1991): N = 14182. Men and women with SCZ, S-AFF or SFD	Compared to general population self-reported rates from National Health Interview Survey (NHIS 1994) – Adams & Marano, 1995	*Field Study*: Self-reported diabetes: lifetime and current. *Medicaid/Medicare Data*: Any diabetes-related claim	*SMI:*<br>(i) Field Study: Lifetime diabetes: 107/719 (14.9%); Current diabetes: 78 cases (10.8%);<br>(ii) Medicaid claims: 673/6066 (11.1%). Age 18-44 = 6.7%; Age 45-64 = 18.8%<br>(iii) Medicare: claims: 1766/14182 (12.5%). Age 18-44 = 5.6%; Age 45-64 = 14.9%<br>*Cited general population rates:*<br>Age 18-44 = 1.2%; Age 45-64 = 6.3%	None reported<br>*Calculated risk ratio = 4.67 (age 18-44 yrs)<br>*Calculated risk ratio = 2.36 (age 45-64 yrs)
Susce et al,2005 (USA) [46]	N=560. Men and women with SCZ, S-AFF, BPAD, DEP	Compared to Kentucky general population (percentages not raw data)	Diagnosis of diabetes (medical chart review)	*SMI:Obs=101/560 (18%)*<br>*Controls = 7%*	Reported OR: 2.9 (95%CI=2.3to 3.6)<br>*Calculated risk ratio=2.57
Inpatient Samples					
Lilliker, 1980 (USA) [26]	N = 1134. Patients with SCZ discharged 1973-1978. No gender information	Compared to general population data from National Health Survey 1960/62	Diabetic diet recorded in dietary records	*SMI:* Obs = 38/1134 (3.3%)<br>*General population rate* = 1.8%	None reported
Regenold et al, 2002 (USA) [27]	N = 71. Men and women with SCZ. Older Adults (age 50-74 yrs)	Compared to general population survey data for age, race and gender matched rates (NHANES III study)	Diagnosis of Diabetes Type 2 or prescription for insulin or oral hypoglycaemics (chart review)	*SMI:* Obs = 9/71 (13%)<br>*General population rate* = 15% (matched controls)	None reported<br>*Calculated risk ratio = 0.84
Hung et al, 2005 (Taiwan) [28]	N = 246. Men and women with SCZ	Compared to general population rates based on data from Lu et al (1998).	Diagnosis of diabetes (on fasting plasma glucose > 126 mg/dL)	*SMI:* Obs = 24/246 (9.8%)<br>*General population:* Obs = 120/1534 (7.8%)	None reported<br>*Calculated risk ratio = 1.25 (0.822 to 1.893)
Regenold et al, 2002 (USA) [27]	N = 20. Men and women with S-AFF. Older Adults (age 50-74 yrs)	Compared to general population survey data (age, race and gender matched rates – NHANES III study)	Diagnosis of Type 2 Diabetes *or* prescription of insulin or oral hypoglycaemics (chart review)	*SMI:* Obs = 10/20 (50%)<br>*General population rate* = 10% (matched controls)	None reported<br>*Calculated risk ratio = 5.0
Cohen et al, 2003 (Netherlands) [29]	N = 93. Men and women with SCZ or S-AFF	Compared to Dutch general population rates (Nivel, 1999 study)	Diabetes Mellitus Type 2 (non-fasting blood glucose > 11.0 mmol/L)	*SMI:* Observed rate = 7.5%<br>*General population rate* = 1.9%	OR = 4.288 (CI 1.979 to 9.289)<br>*Calculated risk ratio = 3.95
Lilliker, 1980 (USA) [26]	*Study 1:* N = 203. Men and women with BPAD. *Study 2:* N = 129. Patients with BPAD discharged 1973-1978. No gender information.	*Study 1:* Compared to general population rates from National Health Survey 1960/62 (N=6692). *Study 2:* Compared to inpatients discharged 1973-1978 with 'other' diagnoses (*including* SCZ).	*Study 1:* Diagnosis of diabetes recorded in notes<br>*Study 2:* Diabetic diet noted in dietary records	*Study 1:* Diagnosed cases (note review):<br>*Bipolar:* Obs = 20/203 (9.85%)<br>Males = 4/79 (5.06%); Females = 16/124 (12.9%)<br>*General population rate (expected)* = 1.8%<br>*Study 2:* Based on dietary records:<br>*Bipolar:* Obs = 16/129 (12.4%)<br>*Other diagnoses:* Obs = 121/4379 (2.8%)	None reported<br>*Calculated risk ratio = 5.47
Cassidy et al, 1999 (USA) [30]	N = 345. Men and women with BPAD	Compared with general population norms	Diagnosis of diabetes (from medical history review) – Type I or Type II	*SMI (matched sample):* Obs = 34/345 (9.9%)<br>*SMI (unmatched sample):* Obs = 36/357 (10.1%)<br>*Expected rate (from US norms)* = 3.5%	None reported<br>*Calculated risk ratio = 2.83

TABLE 1: DIABETES AND HYPERGLYCAEMIA PAPERS (Continued)

Source	Patient sample description	Comparison group description	Outcome measure	Prevalence estimates or Mean/SD	Main results
			Inpatient samples (continued)		
Regenold et al, 2002 (USA) [27]	N = 53. Men and women with BPAD. Older Adults (age 50-74 yrs)	Compared to general population survey data (age, race and gender matched rates – NHANES III study)	Diagnosis of Type 2 Diabetes *or* prescription of insulin or oral hypoglycaemics (chart review)	*SMI:* Obs = 14/53 (26%)<br>*General population rate* = 13% (matched cases)	None reported<br>*Calculated risk ratio = 2.03
Regenold et al, 2002 (USA) [27]	N = 144. Men and women with SCZ, S-AFF or BPAD. Older Adults (age 50-74 years)	Compared to general population survey data (NHANES III study)	Diagnosis of Type 2 Diabetes or prescription for insulin or oral hypoglycaemics (chart review)	*SMI:* Obs = 33/144 (22.9%)<br>*General population rate* = 14%	None reported
Cohen et al, 2003 (Netherlands) [29]	N = 93. Men and women with SCZ or S-AFF	Compared to general population rates – by age group (Hoorn, 1995 study)	Hyperglycaemia (non-fasting blood glucose >7.8 and < 11.0 mmol/L)	*SMI:* Obs = 11/93 (11.83%)<br>Age 20-49 = 15.2%; Age 50-59 = 13.0%<br>*General population rates:*<br>*Age 20-49* = 5.7%; *Age 50-59* = 6.3%	*Age 20-49 years:*<br>OR = 2.959 (CI 1.230 to 7.119)<br>*Age 50-59 years*<br>OR = 2.220 (CI 0.0644 to 7.650)
			Long Stay Samples		
Mukherjee et al, 1996 (Italy) [31]	N = 95. Men and women with SCZ	Compared to general population data from 2 studies – Verriollo et al (1985) and Bruno et al (1992)	Diagnosis of diabetes – record of OGTT fasting plasma glucose > 140 mg/dL on ≥ 2 occasions	*SMI:* Obs = 15/95 (15.8%) (CI 12.1% to 19.5%)<br>*General population rates:*<br>1985 data = 3.2%; 1992 data = 2.1%	None reported<br>*Calculated risk ratio = 4.93
Subramaniam et al, 2003 (Singapore) [32]	N = 194. Men and women with SCZ	Compared to prevalence rates for general population (National Health Survey Singapore 1998)	Prevalence of diabetes (via OGTT using WHO criteria)<br><br>Impaired glucose tolerance (via OGTT using WHO criteria)	*SMI:* Obs = 31/194 (16%)<br>*General population rate* = 9%<br><br>*SMI:* Obs = 60/194 (30.9%)<br>*General population rate* = 15%	None reported<br>*Calculated risk ratio = 1.77<br><br>None reported
			(C) STUDIES COMPARING PATIENTS WITH SMI TO PATIENTS WITH OTHER DIAGNOSES		
			Community Samples		
Chafetz et al, 2005 (USA) [33]	N = 271. Men, women and transgender with SCZ or S-AFF	N = 510. Men and women with 'other' diagnoses (including BPAD)	Diabetes (ascertained from nursing notes)	*SCZ/S-AFF group:* Obs = 20/271 (7.4%)<br>*'Other diagnoses' group:* Obs = 16/510 (3.1%)	None reported<br>*Calculated risk ratio = 2.35 (1.239 to 4.464)
			Outpatient Samples		
Gierz & Jeste, 1993 (USA) [34]	N = 30. Men and women with SCZ – predominantly male. Elderly veterans: mean age 67.6 yrs (SD 6.5)	N = 26. Men and women with DEP from same clinic. Elderly veterans: mean age 66.5 yrs (SD 4.5). Also cite population rates for age >65 years (Schick, 1986).	Diabetes mellitus (ascertained from chart review, computerised profiles, prescription records)	*SCZ group:* Prevalence rate = 16.7%<br>*DEP group:* Prevalence rate = 15.4%<br>*Cited population norms:* Rate = 8.3%.	None reported<br>*Calculated risk ratio = 2.01
Lamberti et al, 2004 (USA) [22]	N = 196. Patients with SCZ. No gender information	N =240. Patients with other psychiatric diagnoses (including S-AFF, BPAD, DEP and other psychotic disorders)	Diagnosis of diabetes (medical chart review)	*Schizophrenia group:* Obs = 27/196 (13.8%)<br>*'Other diagnoses' group:* Obs = 35/240 (14.6%)	None reported
			Inpatient Samples		
Kessing et al, 2004 (Denmark) [35]	N = 6706. Men and women with BPAD. Excluded patients with an *existing* diagnosis of diabetes	(a) N = 108525 patients with osteoarthritis. Men and women<br>(b) N = 29035 patients with DEP. Men and women	Diagnosis of diabetes *during study period:* ICD-8 (Codes 249.00-250.09) or ICD-10 (Codes DE10.0-DE11.9)	*Bipolar:* Obs = 101/6706 (1.51%)<br>*Osteoarthritis:* Obs = 1980/108525 (1.82%)<br>*DEP:* Obs = 358/29035 (1.23%)	None reported<br>*Calculated risk ratio = 1.22 (0.981 to 1.521) – compared to Depression<br>*Calculated risk ratio = 0.83 (0.677 to 1.007) – compared to Osteoarthritis
			Long Stay Samples		
Steinert et al, 1996 (Germany) [36]	N = 90. Men and women with SCZ or S-AFF	N = 90. Men and women with DEP.	Diabetes mellitus (unclear how ascertained from notes)	*SMI:* Males: Obs = 3/43 (7%)<br>Females: Obs = 8/47 (17%)<br>*Non-SMI:* Males: Obs = 2/43 (4.7%)<br>Females: Obs = 3/47 (6.4%)	None reported<br>*Calculated risk ratio = 2.2 (0.796 to 6.076)

Notes: [a] *Adjusted for age and gender;* [b] *Adjusted for age, gender and unemployment;* [c] *Adjusted for body mass index (BMI);* [d] *Adjusted for age, sex and relevant medical risk factors;* [e] *Adjusted for age, gender, year of initial dispensing, ratio of dispensed days to total days, diabetes, anti-angina medications, anti-hypertensive medication;* [f] *Controlling for age, BMI, sex and family history of diabetes*; * If odds ratios or risk ratios are not reported in papers, risk ratios have been calculated wherever possible.

Abbreviations: SCZ = Schizophrenia; S-AFF = Schizoaffective Disorder; BPAD = Bipolar Affective Disorder; N-ACP = Non-affective Chronic Psychotic Illness; SFD = Schizophreniform Disorder; DEP = Depression/Major Depressive Disorder; OGTT = Oral Glucose Tolerance Test. Obs=observations

TABLE 2: HYPERTENSION PAPERS

Source	Patient sample description	Comparison group description	Outcome measure	Prevalence estimates or Mean/SD	Main results
(A) STUDIES WITH A CONTROL GROUP					
Community Samples					
Sokal et al, 2004 (USA)[12]	N = 97. Men and women with SCZ or S-AFF	N = 2861. Matched for age, race and gender (15:1 ratio). Randomly selected from national survey datasets	Lifetime prevalence of hypertension – self-reported in face to face interview	*SMI:* Obs = 24/97 (24.7%)<br>*Control:* Obs = 673/2861 (23.6%)	Unadjusted OR = 1.09 (0.67 to 1.77)<br>Adjusted OR[a] = 1.04 (0.62 to 1.74)<br>Adjusted OR[b] = 1.05 (0.64 to 1.71)<br>*Calculated risk ratio = 1.05 (0.74 to 1.50)
Osborn et al, 2006 (UK: England)[13]c	N = 74. Men and women with SCZ, S-AFF or N-ACP	N = 148. Men and women	Prevalence of hypertension (Systolic BP > 160 or Diastolic BP > 95 mmHg)	*SMI* Obs = 9/74 (12.2%)<br>*Control:* Obs = 19/148 (12.8%)	Unadjusted OR = 0.9 (0.4 to 2.2)<br>Adjusted OR[d] = 0.7 (0.3 to 1.8)<br>Adjusted OR[e] = 0.5 (0.4 to 2.2)<br>*Calculated risk ratio = 0.95 (0.45 to 1.99)
Outpatient and Inpatient Samples					
Curkendall et al, 2004 (USA) [14]	N = 3022. Men and women with SCZ	N = 12088. Men and women. Age and sex matched (4:1 ratio). Randomly selected from health databases	Prevalence of hypertension (ICD-9 Code 401 to 405)	*SMI:* Obs = 414/3022 (13.7%)<br>*Control:* Obs = 2019/12088 (16.7%)	No OR reported<br>*Calculated risk ratio = 0.82 (0.74 to 0.90)
McEvoy et al, 2005 (USA) [47]	N=687. Men and women with chronic SCZ	Compared to population from NHANES III study matched for age/race/gender (n=687)	1) Met metabolic syndrome blood pressure criteria<br>2).Mean systolic BP (mmHg) No SD<br><br>3). Mean diastolic BP (mmHg) No SD	*Male: SMI: 47.2% of 508; controls: 31.1%*<br>*Female: 46.9% of 179; controls: 26.8%*<br>*Male: SMI: 124 mmHg; Control=123.4*<br>*Female: SMI: 122mmHg; Control=119*<br>*Male; SMI: 79 mmHg; Control=77*<br>*Female; SMI: 80mmHg; Control: 73*	No OR Reported; *calculated risk ratio for males and females combined =1.57 (1.37-1.81)<br>SMI=controls T test: p=0.295<br>SMI=controls T test: p=0.063<br>SMI>controls T test: p=0.000<br>SMI>controls T test: p=0.000
Kilbourne et al, 2004 (USA)[16]	N = 4310. Men and women with BPAD or cyclothymia. US Veterans.	N = 3408760. National Veterans cohort. Demographically similar to patient group	Prevalence of hypertension (ICD-9 – codes not specified)	*SMI:* Obs = 1500/4310 (34.8%)<br>*Control:* Obs = 1256034/3408760 (36.8%)	No OR reported<br>*Calculated risk ratio = 0.94 (0.91 to 0.98)
Inpatient Samples					
Saari et al, 2005 (Finland)[20]f	N = 31. Men and women with SCZ. All in early 30's (1966 Birth Cohort)	N = 5455. Men and women from same birth cohort.	Prevalence of hypertension (BP ≥ 130/85 mmHg)	*SMI:* Obs = 15/31 (48%)<br>*Control:* Obs = 2209/5455 (40%)	No OR reported<br>*Calculated risk ratio = 1.19 (0.83 to 1.72)
(B) STUDIES COMPARING PATIENTS WITH SMI TO NORMATIVE DATA					
Outpatient Samples					
Dickerson et al, 2002 (USA)[23]	N = 43. Females with SCZ or S-AFF. Age 40-70 years.	N = 101. Age-matched females from Maryland Behavioural Risk Factor Survey (BFRS) 1999 dataset	Prevalence of hypertension – self-reported in interview (using BFRS)	*SMI:* Obs = 16/43 (37%) (CI: 7%-53%)<br>*General population rate* = 28% (CI: 24%-31%)	No OR reported<br>*Calculated risk ratio = 1.33
Outpatient and Inpatient Samples					
Casadebaig et al, 1997 (France)[24]	N = 3470. Men and women with SCZ	Men and women from INSEE-CREDES 1991 survey dataset (representative sample of French general population)	Prevalence of hypertensive disease (ICD-9 Codes 401-405)	*SMI Males:* Obs = 51 cases<br>*SMI Females:* Obs = 33 cases<br>Comparison group rates not reported	Standardised Morbidity Ratios (no CI reported): Male SMR = 2.0; Female SMR = 0.5
Cohn et al, 2004 (Canada)[37]f	N = 240. Men and women with SCZ or S-AFF	General population rates from Canadian Heart Health Survey (1986-1990)	Prevalence of hypertension (BP ≥ 135/85 mm Hg *or* current treatment with antihypertensive medication)	Presented as graphs – exact values not given in table or text.	No OR reported
Susce et al,2005 (USA) [46]	N=560. Men and women with SCZ, S-AFF, BPAD, DEP	Compared to Kentucky general population prevalence; no raw data	Prevalence of hypertension from medical charts	SMI: obs 151/560= 27%<br>Control = 30%	OR=0.86,95%CI=0.71 to 1.03)<br>*Calculated risk ratio=0.9

TABLE 2: HYPERTENSION PAPERS (continued)

Source	Patient sample description	Comparison group description	Outcome measure	Prevalence estimates or Mean/SD	Main results
			Inpatient Samples		Patie
Yates & Wallace, 1987 (USA) [38]c	N = 50. Men and women with BPAD. Study <u>excluded</u> non-white patients	Expected rates derived from general population data for age- and gender-specific controls (NHANES II Survey data 1971-75).	Prevalence of hypertension – defined as: (1) Patient history or physician diagnosis of hypertension; (2) Use of antihypertensive medication; (3) mean BP > 160/95 mmHg<br><br>Also report mean systolic and diastolic BP (with 95% CI)	Obs = 14/50 (28%); Exp = 5.6 (11.2%)<br>*Males:* Obs = 7/25 (28%); Exp = 3.0 (12%)<br>*Females:* Obs = 7/25 (28%); Exp = 2.6 (10.4%)<br><br>Mean SBP = 128.7 (124.1-132.33)<br>*Males* = 123.7 (117.3-130.00)<br>*Females* = 133.8 (127.8-139.0)<br>Mean DBP = 81.0 (77.9-84.1)<br>*Males* = 79.5 (75.4-83.6)<br>*Females* = 82.5 (77.6-87.4)	None reported<br>*Calculated risk ratio = 2.5
			Long Stay Samples		
Steinert et al, 1996 (Germany) [36]c	N = 90. Men and women with SCZ or S-AFF. Age range 41-90 years	Data from German DHP Studie (1988) – Men and women, aged 40-70 years.	Mean systolic and diastolic blood pressure (with SD)	*SMI:* Male = 126.8 (11.3)/79.3 (7.1)<br>Female = 127.2 (15.4)/78.2 (5.9)<br>*General population data:*<br>Male = 139.2 (19.5)/85.4 (11.9)<br>Female = 136.0 (15.8)/82.0 (11.2)	None reported
			(C) STUDIES COMPARING PATIENTS WITH SMI TO PATIENTS WITH OTHER DIAGNOSES		
			Community Samples		
Chafetz et al, 2005 (USA) [33]	N = 271. Men and women with SCZ or S-AFF	N = 510. Men and women. Patients with other psychiatric diagnoses	Prevalence of hypertension (derived from nurse records)	*SMI:* Obs = 39/271(14.4%)<br>*Other diagnoses:* Obs = 56/510 (11.0%)	No OR reported<br>*Calculated risk ratio = 1.31 (0.90 to 1.92)
			Outpatient Samples		
Gierz & Jeste, 1993 (USA) [34]	N = 30. Elderly men and women with SCZ. From US Veterans clinic: mean age 67.6 yrs (SD 6.5)	N = 26. Elderly men and women with DEP from same clinic: mean age = 66.5 yrs (SD 4.5)	Prevalence of hypertension (derived from chart/records review)	*SMI:* Observed rate = 26.7%<br>*DEP:* Observed rate = 46.2%<br>*General population rate:* Over 65 years = 37.9%	No OR reported<br>*Calculated risk ratio = 0.58 (vs Depression)<br>*Calculated risk ratio = 0.70 (vs gen popn)
			Long Stay Samples		
Steinert et al, 1996 (Germany) [36]c	N = 90. Men and women with SCZ or S-AFF. Age range = 41-90 years	N = 90. Men and women with DEP. Age range 39-88 years	Prevalence of hypertension – definition unclear. Also report mean systolic and diastolic blood pressure (with SD)	*SMI*: Males – Obs = 3/43 (7%)<br>Females – Obs = 5/47 (10.6%)<br>*DEP:* Males – Obs = 2/43 (4.7%)<br>Females – Obs = 2/47 (4.3%)<br><br>Mean SBP/DBP<br>*SMI:* Male = 126.8 (11.3)/79.3 (7.1)<br>Female = 127.2 (15.4)/78.2 (5.9)<br>*DEP:* Male = 123.2 (15.3)/76.7 (8.5)<br>Female = 124.6 (15.9)/78.0 (9.3)	None reported.<br>*Calculated risk ratio = 2.00 (0.62 to 6.41)

Notes on Tables: [a] Adjusted for BMI; [b] Adjusted for smoking; [c] Study also reports mean systolic and diastolic BP values; [d] Adjusted for age and gender; [e] Adjusted for age, gender and unemployment; [f] Data presented as part of metabolic syndrome study. * If odds ratios or risk ratios are not reported in papers, risk ratios have been calculated wherever possible.

Abbreviations: SCZ = Schizophrenia; S-AFF = Schizoaffective Disorder; BPAD = Bipolar Affective Disorder; N-ACP = Non-affective Chronic Psychotic Illness; SFD = Schizophreniform Disorder; DEP = Depression/Major Depressive Disorder. BP: Blood presure

TABLE 3: DYSLIPIDEMIA PAPERS

Source	Patient sample description	Comparison group description	Outcome measure	Prevalence estimates or Mean/SD	Main results
(A) STUDIES WITH A CONTROL GROUP					
Community Samples					
Osborn et al, 2006<br>(UK: England) [13]c	N = 74. Men and women with SCZ, S-AFF or N-ACP	N = 148. Men and women	Prevalence of raised total cholesterol (> 5.1 mmol/L)	*SMI:* Obs = 41/74 (56.2%)<br>*Control:* Obs = 73/148 (49.3%)	Unadjusted OR = 1.3 (0.7 to 2.5)<br>Adjusted OR = 1.4 (0.8 to 2.5) [b]<br>Adjusted OR = 1.9 (0.9 to 3.9) [c]
			Prevalence of low HDL cholesterol (< 1.0 mmol/L)	*SMI:* Obs = 12/74 (16.7%)<br>*Control:* Obs = 7/148 (4.8%)	Unadjusted OR = 4.0 (1.5 to 10.7)<br>Adjusted OR = 3.9 (1.4 to 10.8) [b]<br>Adjusted OR = 2.2 (0.7 to 7.6) [c]
			Prevalence of high total/HDL cholesterol ratio	*SMI:* Obs = 43/74 (59.7%)<br>*Control:* Obs = 66/148 (44.9%)	Unadjusted OR = 1.8 (1.0 to 3.2).<br>Adjusted OR = 1.7 (0.9 to 3.0) [b]<br>Adjusted OR = 1.3 (0.7 to 2.6) [c]
			Mean total cholesterol (mmol/L)	*SMI*: Mean = 5.41 (SD 1.3)<br>*Control:* Mean = 5.3 (SD 1.3)	*Standardised mean diff= 0.08 (-0.20 to 0.36)
			Mean HDL cholesterol (mmol/L)	*SMI:* Mean = 1.4 (SD 0.45)<br>*Control:* Mean = 1.6 (SD 0.48)	*Standardised mean diff = -.42 (-0.71 to -0.14)
			Mean LDL cholesterol (mmol/L)	*SMI:* Mean = 2.98 (SD 1.05)<br>*Control:* Mean = 2.98 (1.12)	*Standardised mean diff = 0(-0.30 to 0.30)
			Mean triglycerides (mmol/L)	*SMI:* Mean = 2.5 (SD 1.7)<br>*Control:* Mean = 1.8 (SD 1.5)	*Standardised mean diff = 0.47(0.16 to 0.73)
			Mean total/HDL cholesterol ratio	*SMI:* Mean = 4.3 (SD 1.5)<br>*Control:* Mean = 3.7 (SD 1.3)	*Standardised mean diff = 0.44 (0.15 to 0.72)
McEvoy et al, 2005<br>(USA) [47]	N=687. Men and women with chronic SCZ	Compared to population from NHANES III study matched for age/race/gender (n=687)	Met metabolic syndrome criteria for HDL cholesterol (mg/dl)	*Males; SMI 48.9%, Control 31.9%*<br>*Females SMI 63.3% Controls 36.3%*	SMI>Controls Chi Sq P=0.000<br>SMI>controls Chi Sq P=0.001
			Met metabolic syndrome triglyceride (mg/dl) criteria	*Males: SMI= 50.7% Contols=32.1%*<br>*Females; SMI=42.3% Controls=19.6%*	SMI>controls Chi Sq P=0.000<br>SMI>controls Chi Sq P=0.000
			Mean HDL cholesterol (mg/dl) [fasting]	*Males: SMI= 42.3 mg/dl; Controls=47.2 (No SD)*<br>*Females: SMI= 47.7; Controls: 55.2 (No SD)*	SMI<controls T test P=0.000<br>SMI<controls T test P=0.000
			Mean triglycerides (mg/dl) [fasting]	*Males: SMI=194.7 Conrtols=143.6 (No SD)*<br>*Females: SMI=173.8, Controls=118.9 (No SD)*	SMI>controls T test P=0.000<br>SMI>controls T test P=0.000
Outpatient and Inpatient Samples					
Curkendall et al, 2004<br>(USA)[14]	N = 3022. Men and women with SCZ	N = 12,088. Age and sex matched to patients (4:1 ratio). Randomly selected from health databases	Prevalence of disorders of lipid metabolism (ICD-9 Code 272) *or* claims for antilipemic medication	*SMI:* Rate = 5.4%<br>*Control:* Rate = 6.3%	No OR reported<br>*calculated risk ratio 0.86 (0.73, 1.01)
Kilbourne et al, 2004<br>(USA)[16]	N = 4310. Men and women with BPAD. US Veterans.	N = 3408760. National Veterans cohort. Demographically similar to patient group.	Prevalence of hyperlipidemia (ICD-9, codes not specified)	*SMI:* Obs = 973/4310(22.6%)<br>*Control:* No lipid data presented	No OR reported

TABLE 3: DYSLIPIDEMIA PAPERS (continued)

Source	Patient sample description	Comparison group description	Outcome measure	Prevalence estimates or Mean/SD	Main results
Inpatient Samples					
Saari et al, 2005 (Finland) [20]d	N = 31. Men and women with SCZ. All in early 30's (1966 Birth Cohort followed up 1997/98)	N = 5455. Men and women from same birth cohort without history of psychiatric treatment	Prevalence of low HDL cholesterol (< 40 mg/dL in men; < 50 mg/dl in women)	*SMI:* Obs = 5/31(16%)<br>*Control:* Obs = 588/5455 (11%)	No OR reported<br>*calculated risk ratio 1.50 (0.66, 3.39)
Saari et al, 2004 (Finland) [39]	N = 31. Men and women with SCZ. All age 31 (1966 birth cohort followed up in 1997).	N = 5498. Men and women from same birth cohort without history of psychiatric treatment	Mean total cholesterol (mg/dL)	*SMI:* Mean = 214.1 mg/dL (SD 33.6)<br>*Control:* Mean = 196.4 mg/dL (SD 39.0)	ANOVA[e]: SMI > Controls (p = .039)<br>*Standardised mean difference = 0.46 (0.10 to 0.81)
			Mean HDL cholesterol (mg/dL)	*SMI:* Mean HDL = 55.9 mg/dL (no SD)<br>*Control:* Mean HDL = 60.5 mg/dL (no SD)	ANOVA[e]: SMI = Controls (n/sig)
			Mean LDL cholesterol (mg/dL)	*SMI:* Mean = 131.5 mg/dL (SD 30.5)<br>*Control:* Mean = 116.3 mg/dL (no SD)	ANOVA[e]: SMI = Controls (n/sig)
			Mean fasting triglycerides (mg/dL)	*SMI:* Mean = 134.9 (no SD)<br>*Control:* Mean = 104.3 (no SD)	ANOVA[e]: SMI > Controls (p = .028)
Ryan et al, 2003 (UK/Eire) [19]	N = 26. Men and women with SCZ. All first-episode and drug-naïve. Mean age 33.6 years	N = 26. Men and women. Mean age 34.4 years. Matched for age, exercise, diet, smoking, alcohol intake and anthropometric measures.	Mean fasting total cholesterol (mmol/L)	*SMI:* Mean = 4.02 mmol/L (SD 0.78)<br>*Control:* Mean = 4.57 mmol/L (SD 0.81)	T-test: SMI < Controls (p <.02)<br>*Standardised mean difference = -0.69 (-1.25 to -01.3)
			Mean HDL cholesterol (mmol/L)	*SMI:* Mean = 1.20 mmol/L (SD 0.44)<br>*Control:* Mean = 1.25 mmol/L (SD 0.25)	T-test: SMI = Controls (n/sig)<br>*Standardised mean difference = -0.14 (-0.68 to 0.40)
			Mean fasting LDL cholesterol (mmol/L)	*SMI:* Mean = 2.39 mmol/L (SD 0.84)<br>*Control:* Mean = 2.91 mmol/L (SD 0.69)	T-test: SMI < Controls (p <.02)<br>*Standardised mean difference = -0.68 (-1.24 to -0.12)
			Mean fasting triglycerides (nmol/L)	*SMI:* Mean = 0.99 mmol/L (SD 0.43)<br>*Control:* Mean = 0.92 mmol/L (SD 0.30)	T-test: SMI = Controls (n/sig)
Scottish Schizophrenia Research Group, 2000 (UK: Scotland) [40]	N = 30. Men and women with SCZ or SFD. First episode, drug-naïve. Mean age 28 years (males)/33 years (females)	N = 30. Matched for gender and age, smoking and dietary status. Mean age 30 years	Mean serum cholesterol (mmol/L)	*SMI:* Mean = 4.63 mmol/L (SD 0.80)<br>*Control:* Mean = 4.98 mmol/L (SD 0.91)	SMI = Controls (n/sig)<br>*Standardised mean difference = -0.41 (-0.92 to 0.10) – note: 73% sample smoked
			Mean serum lipid peroxide levels (umol/L)	*SMI:* Mean = 0.50 umol/L (SD 0.16)<br>*Control:* Mean = 0.54 umol/L (SD 0.23)	SMI = Controls (n/sig)<br>(Note: 73% sample smoked)

TABLE 3: DYSLIPIDEMIA PAPERS (Continued)

Source	Patient sample description	Comparison group description	Outcome measure	Prevalence estimates or Mean/SD	Main results
(B) STUDIES COMPARING PATIENTS WITH SMI TO NORMATIVE DATA					
Outpatient and Inpatient Samples					
Cohn et al, 2004 (Canada) [37]d	N = 240. Men and women with SCZ or S-AFF	General population rates from anadian Heart Health Survey (1986-1990)	Mean total cholesterol (mmol/L)	Means presented as graphs – exact values not given in table or text	T-tests: SMI = Controls (n/sig) for males and females
			Mean HDL cholesterol (mmol/L)	Means presented as graphs – exact values not given in table or text	T-tests: SMI < Controls for males (p > .001) and females (p = 002)
			Mean triglycerides (mmol/L)	Exact means/SDs not specified in text or a table. Means presented as graphs (with error bars)	T-tests: SMI > Controls for males (p = .001) and females (p = .009)
Long Stay Samples					
Steinert et al, 1996 (Germany) [36]	N = 90. Men and women with SCZ or S-AFF. Age range = 41-90	General population survey data (DHP Study 1988)	Mean total cholesterol (mg/dL)	*SMI:* Males: Mean = 205.1 mg/dL (SD 37.0)<br>Females: Mean = 201.7 mg/dL (SD 44.2)<br>*General population:*<br>Males: Mean = 242.9 mg/dL (SD 45.0)<br>Females: Mean = 250.7 mg/dL (SD 45.1)	SMI < Controls for males and females (ps <.001)
(C) STUDIES COMPARING PATIENTS WITH SMI TO PATIENTS WITH OTHER DIAGNOSES					
Inpatient Samples					
Yates & Wallace, 1987 (USA) [21]	N = 50. Men and women with BPAD. *Excluded* non-white patients	N = 50. Men and women with DEP, matched for gender and age	Mean fasting cholesterol (mg/dL). *Normal range* cited as 130-315 mg/dL	*SMI:* Mean = 184.5 mg/dL (CI: 173.0 to 196.0)<br>Males: Mean = 180.4 (CI 164.2 to 196.6)<br>Females: Mean = 189.0 (CI 171.6 to 206.4)<br>*DEP:* Mean = 204.3 mg/dL (No CI's reported)<br>Males: Mean = 198.2; Females: Mean = 210.4	None reported
Long Stay Samples					
Steinert et al, 1996 (Germany) [36]	N = 90. Men and women with SCZ or S-AFF. Mean age 62.6 years (range 41-90)	N = 90. Men and women with DEP. Mean age 62.3 years (range 39-88).	Mean total cholesterol (mg/dL)	*SMI:* Males: Mean = 205.1 mg/dL (SD 37.0)<br>Females: Mean = 201.7 mg/dL (SD 44.2)<br>*DEP:* Males: Mean = 219.7 mg/dL (SD 50.6)<br>Females: Mean = 223.5 mg/dL (SD 42.9)	None reported
			Mean triglycerides (mg/dL)	*SMI:* Males: Mean = 128.9 mg/dL (SD 72.6)<br>Females: Mean = 137.2 mg/dL (SD 70.9)<br>*DEP:* Males: Mean = 158.0 mg/dL (SD 77.8)<br>Females: Mean = 136.8 mg/dL (SD 69.5)	None reported
Source of sample unclear					
Roccatagliata et al, 1980 (Italy) [41]	N = 60. Men and women with SCZ.	N = 60. Men and women with neurological or other psychiatric disorders. Matched for age and sex.	Mean total cholesterol (mg/dL)	*SMI:* Mean = 188.2 mg/dL (SD 45.4)<br>*Other diagnosis:* Mean = 219.0 mg/dL (SD 31.8)	T-test: SCZ < Controls (p = .005)
			Mean total lipid (mg/dL)	*SMI:* Mean = 821.5 mg/dL (SD 539.9)<br>*Other diagnosis:* Mean = 840.1 mg/dL (SD 157.7)	T-test: SCZ = Controls (n/sig)
			Mean triglycerides (mg/dL)	*SMI:* Mean = 173.3 mg/dL (SD 306.7)<br>*Other diagnosis:* Mean = 131.2 mg/dL (SD 45.4)	T-test: SCZ = Controls (n/sig)

Notes on Table: [a] *Osborn et al (2006) also report mean total/HDL cholesterol ratios and mean values (mmol/L) for total cholesterol, HDL cholesterol, LDL cholesterol and triglycerides.* [b] *Adjusted for age and gender:* [c] *Adjusted for age, gender and unemployment.* [d] *Data presented as part of metabolic syndrome study.* [e] *Adjusted for sex, BMI, smoking and alcohol consumption.* * If odds ratios or risk ratios are not reported in papers, risk ratios or standardised mean differences have been calculated wherever possible.

Abbreviations: SCZ = Schizophrenia; S-AFF = Schizoaffective Disorder; BPAD = Bipolar Affective Disorder; N-ACP = Non-affective Chronic Psychotic Illness; SFD = Schizophreniform Disorder; DEP = Unipolar Depression/Major Depressive Disorder

TABLE 4: METABOLIC SYNDROME PAPERS

Source	Patient sample description	Comparison group description	Outcome measures	Prevalence estimates	Main results
(A) STUDIES WITH A CONTROL GROUP					
Inpatient Samples					
Saari et al, 2005 (Finland) [20]	N = 31. Men and women with SCZ. All in early 30's (from North Finland 1966 Birth Cohort followed up 1997/98).	N = 5455. Men and women from same 1966 birth cohort with no history of psychiatric treatment.	Prevalence of metabolic syndrome – defined as the presence of 3 or more of 5 NCEP ATP III criteria [a]	*SMI:* Obs = 6/31 cases (19.4%)<br>*Control:* Obs = 326/5455 cases (6%)	Adjusted OR = 3.7 (CI: 1.5 to 9.0) [b]<br>*Calculated risk ratio = 3.24 (1.57 to 6.69)
McEvoy et al, 2005 (USA) [47]	N=687. Men and women with chronic SCZ	Compared to population from NHANES III study matched for age/race/gender (n=687)	Prevalence of metabolic syndrome – defined as the presence of 3 or more of 5 NCEP ATP III criteria [a]	*Male SMI: 183/508 (36.0%) Controls: 19.7%*<br>*Female SMI: 92/178 (51.6%) Controls: 25.1%*	No OR reported.<br>*calculated risk ratios:<br>Male: 1.83 (1.42-2.36)<br>Female: 2.02 (1.40-2.96)
(B) STUDIES COMPARING PATIENTS WITH SMI TO NORMATIVE DATA					
Outpatient Samples					
Heiskanen et al, 2003 (Finland) [42]	N = 35. Men and women with SCZ or S-AFF	Finnish general population data from Vanhala et al (1997) and Laaksonen et al (2002) studies	Prevalence of metabolic syndrome – defined as the presence of 3 or more of 5 NCEP ATP III criteria [a]	*SMI:* Obs = 13/35 cases (37%)<br>Males = 9/19 (47%); Females = 4/16 (25%)<br>*General population rates:*<br>Males = 11-17%; Females = 6-20%	None reported<br>*Calculated risk ratio = 3.38 (males)<br>*Calculated risk ratio = 2.00 (females)
Basu et al, 2004 (USA) [43]	N = 33. Men and women with S-AFF	Data from US epidemiological study (N=8814) – Ford et al 2002	Prevalence of metabolic syndrome – defined as the presence of 3 or more of 5 NCEP ATP III criteria [a]	*SMI:* Obs = 14/33 cases (42.4%)<br>Males = 7/14 (50.0%); Females = 7/19 (36.8%)<br>*General population rate* = 23.7%	None reported<br>*Calculated risk ratio = 1.79
Outpatient and Inpatient Samples					
Cohn et al, 2004 (Canada) [37]	N = 240. Men and women with SCZ or S-AFF	Data from US epidemiological study (N=8814) – Ford et al 2002	Prevalence of metabolic syndrome – defined as the presence of 3 or more of 5 NCEP ATP III criteria [a] and/or current treatment with anti-hypertensive or anti-diabetic medication	*SMI rates:*<br>Males = 42.6%; Females = 48.5%<br>*General population rates:*<br>Males = 24%; Females = 23%	None reported<br>*Calculated risk ratio = 1.77 (males)<br>*Calculated risk ratio = 2.11 (females)

Note: [a] *The National Cholesterol Education Program (NCEP) ATP III criteria for Metabolic Syndrome are: (1) Abdominal obesity – waist circumference > 102 cm (40 inc) in men; > 88 cm (35 inc) in women; (2) Fasting hypertriglyceridemia - ≥ 1.69 mmol/L or 150 mg/dL; (3) Low fasting HDL - < 1.04 mmol/L or 40 mg/dL in men; <1.29 mmol/L or 50 mg/dL in women; (4) Hypertension - ≥ 130/85 mmHg; (5) Fasting Hyperglycaemia - ≥ 6.1 mmol/L or 110 mg/dL.* [b] *Odds ratio adjusted for gender.* * If odds ratios or risk ratios are not reported in papers, risk ratios have been calculated wherever possible.

Abbreviations: SCZ = Schizophrenia; S-AFF = Schizoaffective Disorder; BPAD = Bipolar Affective Disorder.

TABLE 5: FRAMINGHAM RISK SCORE PAPERS

Source	Patient sample description	Comparison group description	Outcome measure	Prevalence estimates or Mean/SD or Median/IQR	Main results
(A) STUDIES WITH A CONTROL GROUP					
Community Samples					
Osborn et al, 2006 (UK: England) [13]	N = 74. Men and women with SCZ, S-AFF or N-ACP	N = 148. Men and women.	Prevalence of raised Framingham Risk Score for CHD (higher than expected for individual's age and gender)<br>Median absolute 10-year Framingham Risk Score (%) for CHD, with interquartile range<br>Mean Framingham Risk Score excess for CHD	*SMI:* Obs = 37 cases (51.4%)<br>*Control:* Obs = 55 cases (37.4%)<br>*SMI:* Median = 5%; IQR = 2-12%<br>*Control:* Median = 4%; IQR = 2-9%<br>*SMI:* Mean = 1.99 (SD 7.0)<br>*Control*: Mean = 0.69 (SD 4.6)	Unadjusted OR = 1.7 (1.0 to 3.1)<br>Adjusted OR = 1.7 (0.9 to 3.0) [a]<br>Adjusted OR = 1.3 (0.7 to 2.7) [b]<br>Mann-Whitney test: SMI > Controls (p = .049)<br>T-test: SMI = Controls (n/sig)<br>*Standardised mean difference = 0.219 (-0.0441 to 0.516)
Inpatient Samples					
Luty et al, 2002 (UK: Scotland) [44]	N = 21. Men and women with SCZ or SFD. Mean age 31 years. First episode of illness; neuroleptic naïve	N = 25. Men and women. Mean age 30 years. Matched for gender, age, smoking and dietary status	Mean (10-year) Framingham Risk Score (%) for Heart Disease [c]	*SMI (9 matched pairs):* Mean score = 6% (SD 5)<br>*Control (9 matched pairs):* Mean score = 4% (SD 5)<br>*General population data:* Mean score = 2% (SD 3).	T-tests: SMI=Controls (n/sig); SMI>gen popn (p=.008); Controls>gen popn (p = .02)<br>*Standardised mean differences:<br>SMI vs matched controls = 0.4 (-0.186 to 0.986)<br>SMI vs general population = 0.97
(B) STUDIES COMPARING PATIENTS WITH SMI TO NORMATIVE DATA					
Community Samples					
McCreadie, 2003 (UK: Scotland) [45]	N = 102. Men and women with SCZ	Compared with general population norms (Scottish Health Survey, 1998)	Mean (10-year) Framingham Risk Score (%) for CHD [c]	*SMI:* Males: Mean score = 10.5% (SD 8)<br>Females: Mean score = 7% (SD 6)<br>*General population norms*<br>Males: Mean score = 6.4% (SD 6)<br>Females: Mean score = 4.1% (SD 4)	T-tests: Males: SMI>gen popn (p = .001); Females: SMI=gen popn (p = .06)<br>*Standardised mean differences:<br>= 0.58 (males); = 0.568 (females)
Outpatient and Inpatient Samples					
Cohn et al, 2004 (Canada) [37]	N = 240. Men and women with SCZ or S-AFF	N = 7020. Men and women, randomly selected from Canadian Heart Health Survey dataset (1986-1990). Matched for age and gender distribution.	Mean (10-year) Framingham Risk Score (%) for myocardial infarction	*SMI:* Males*:* Mean score = 8.9% (no SD)<br>Females: Mean score = 2.6% (no SD)<br>*General population:*<br>Males: Mean score = 6.3% (no SD)<br>Females: Mean score = 2.0% (no SD)	T-tests: Males: SMI > General population (p <.001); Females: SMI = Controls (n/sig)

Notes: [a] *Adjusted for age and gender.* [b] *Adjusted for age, gender and unemployment.* [c] *Also report Framingham Risk Score for Stroke.* * Standardised mean differences have been calculated where possible.

Abbreviations: SCZ = Schizophrenia; S-AFF = Schizoaffective Disorder; N-ACP = Non-Affective Chronic Psychotic Illness; SFD = Schizophreniform Disorder
